# Histology-based prediction of lymph node metastases in early gastric cancer as decision guidance for endoscopic resection

**DOI:** 10.18632/oncotarget.7221

**Published:** 2016-02-06

**Authors:** Ulrich Ronellenfitsch, Christiane Lippert, Rainer Grobholz, Siegfried Lang, Stefan Post, Georg Kähler, Timo Gaiser

**Affiliations:** ^1^ Department of Surgery, University Medical Center Mannheim, University of Heidelberg, Mannheim, Germany; ^2^ Institute of Pathology, University Medical Center Mannheim, University of Heidelberg, Mannheim, Germany; ^3^ Institute of Pathology, Kantonsspital Aarau, Aarau, Switzerland; ^4^ First Department of Medicine, Division of Biostatistics, University Medical Center Mannheim, University of Heidelberg, Mannheim, Germany

**Keywords:** early gastric cancer, lymph node metastases, histopathological risk factors, endoscopic treatment, Western study population

## Abstract

**Background:**

Selected cases of early gastric cancer (EGC) can be successfully treated by endoscopic therapy if the risk of concurrent lymph node metastases (LNM) is negligible. Criteria for endoscopic resection based on risk factor analyses for LNM have been established mainly in Asia. However, it is not clear to what extent these recommendations can be transferred to Western collectives. The aim of this study was to analyze predictors for LNM in EGC in a Western study population.

**Methods:**

From our institutional archive, we selected all patients with gastric adenocarcinoma who had undergone gastrectomy with lymphadenectomy (1972 – 2005). Among 1970 patients 275 cases with EGC were identified. Clinical and pathological data were collected and logistic regression analyses performed.

**Results:**

LNM were present in 36/275 (13.1%) patients. With deeper invasion proportion of LNM increased. At submucosa level (sm1), patients were almost five times more likely to have LNM than at mucosa levels.

Multivariable logistic regression analysis revealed lymphovascular invasion, diffuse- and mixed-type, and invasion depth as significant independent histopathological predictors of LNM. In patients with intestinal type according to Lauren and no lymphovascular invasion, we found only one LNM-positive case out of 43 patients in the pT1b (sm1 and sm2) groups.

**Conclusions:**

Our results underline the recommendation of most guidelines that endoscopic resection is sufficient for pT1a ECG because of the low incidence of LNM in this group. However, there seems also a role for endoscopic therapy in cases of pT1b (sm1/2) EGC with intestinal type differentiation and no lymphovascular invasion.

## INTRODUCTION

Despite a decline in incidence, gastric cancer is still the fourth most common cause of cancer-related mortality in Western countries [1]. As advanced stages are present in more than half of patients, the generally poor prognosis is only improved by timely diagnosis of early gastric cancers (EGC). According to the WHO classification, these are defined as adenocarcinomas of the stomach restricted to the mucosa or submucosa (pT1a or pT1b), regardless of whether lymph node metastases (LNM) are present or not [1, 2]. However, the prognosis of EGC is excellent with a more than 90% five-year survival rate only in cases without LNM [3].

As treatment options for EGC, procedures such as endoscopic mucosal resection and endoscopic submucosal dissection are considered to be efficient and minimally invasive approaches. However, as lymph nodes cannot be removed endoscopically, both techniques should only be applied in the absence of LNM [4-7]. Therefore, accurate staging and prediction of the risk of LNM are crucial for selecting suitable patients for endoscopic procedures. Unfortunately, there is no wholly reliable upfront staging method for identifying LNM. In several studies, computed tomography, endoscopic ultrasonography and positron emission tomography failed to predict the presence of LNM accurately [8-10]. The current gold standard for assessing the risk of LNM is therefore based on histological and clinical features of the primary tumor after endoscopic resection. Several histological and clinical factors have been shown to be associated with the occurrence of LNM in ECG: e.g. macroscopic tumor type, tumor size, tumor location, depth of invasion, presence of vascular or lymphatic invasion and histological classification [11-13]. These findings resulted in treatment guidelines recommending endoscopic procedures for mucosal and at least partly for submucosal tumors. However, the exact criteria which carcinoma can still be treated with endoscopic resection are unclear and recommendations differ between guidelines.

Guidelines from the National Comprehensive Cancer Network (NCCN) restrict endoscopic treatment to pT1a tumors with a diameter ≤2 cm, good or moderate tumor differentiation, no ulceration and no lymphovascular invasion [14]. The clinical practice guidelines of the European Society for Medical Oncology (ESMO) give similar recommendations regarding tumor size, depth of invasion and absence of ulceration but restrict endoscopic treatment to well differentiated tumors and furthermore do not appreciate lymphovascular invasion as a risk factor for LNM [15]. The treatment guidelines of the Japanese Gastric Cancer Association (JGCA) consider endoscopic resection still curative in pT1b EGC if the tumor has a diameter of ≤ 3 cm, is of differentiated histology (papillary or tubular adenocarcinoma) and has a submucosal infiltration depth of < 500 μm [16].

The basis of all of these recommendations is a risk estimate of concurrent LNM in EGC. If the incidence of LNM with certain clinical and pathological factors is estimated to be very low, lymphadenectomy is not required and endoscopic treatment is regarded curative [17]. However, these risk estimates require large studies to accrue a sufficient number of patients in each group, and are therefore very difficult to perform due to the general small number of EGC patients. Nearly all studies achieving these numbers were performed in Asian populations, where EGC is much more prevalent and endoscopic screening and treatment is more widely performed than in Europe and North America. Since differences in biology and aetiology of gastric cancer might exist between Eastern and Western countries, it could be inappropriate to transfer Eastern treatment recommendations without further examination in Western collectives [10, 18].

The few available smaller Western studies could already identify a number of risk factors in EGC for the incidence of LNM [19-23]. Among those were tumor size, Lauren type, tumor differentiation, macroscopic aspect of the tumor, and invasion depth. However, risk factors were inconsistently identified across studies, and only one of the series assessed lymphovascular invasion as a potential risk factor [19]. Consequently, it remains unclear if Eastern criteria for endoscopic treatment can be safely applied in this population. Therefore, we recognized the need to precisely identify histopathological predictors of LNM in EGC in a large Western patient population.

## MATERIALS AND METHODS

### Patients and data collection

This retrospective study comprised 275 consecutive patients with histologically confirmed pT1 gastric adenocarcinoma who underwent surgical resection (total or subtotal gastrectomy) including systematic lymphadenectomy between October 1972 and June 2005 at the University Medical Center Mannheim, Germany. After this date, definite endoscopic treatment became the institutional standard for select cases of EGC. In total, 1,970 patients with gastric adenocarcinoma were operated at the institution during the study period. None of the patients had received neoadjuvant chemotherapy. Clinical data were gathered retrospectively from an institutional database. Primary tumors were cut into 2-mm serial sections followed by complete histopathological examination. Data about depth of tumor invasion, tumor size, lymphovascular invasion, Lauren classification and LNM were reanalysed by experienced gastrointestinal pathologists (RG, TG), respectively. The Lauren classification was established with the two major histological subtypes (intestinal type and diffuse type) and mixed type. Histologically, intestinal-type carcinomas were diagnosed on the basis of cohesive, gland-forming tumor cells with expanding or infiltrative growth patterns, whereas diffuse-type carcinomas showed non-cohesive, non-gland-forming tumor cells with diffuse growth patterns. Mixed type was diagnosed if a combination of the two components was present independently of their distribution or proportion. Primary tumors (T category) were classified by applying the seventh version of the UICC TNM staging system of gastric cancer [24]. Depth of invasion was classified according to which third of the mucosa or submucosa was reached (m1 to 3, sm1 to 3). Tumor size was determined as the maximal diameter in any dimension on formalin fixed specimens. The study was approved by the institutional ethics committee (reference number 805R/2014).

### Statistical analyses

For univariable analyses, patients were divided into those with and those without LNM in final histopathological work-up. For data with normal distribution, the Student's *t*-test was applied to compare the two groups. For non-normally distributed data, the Mann-Whitney *U* test was used as a non-parametric test. Deviations from the Gaussian distribution were tested by the Kolmogorov-Smirnov test. Non-continuous (categorical) variables were analysed by use of a 2×2 table, Fisher's exact test and the chi-squared test. To identify independent predictors of LNM, multivariable logistic regression analysis was performed with LNM as the dependent binary variable and gender, age, depth of invasion, lymphatic invasion and subtype according to the Lauren classification as independent variables. There was no imputation of missing information for single patients. A *p*-value of *p*≤0.05 (two-tailed) was considered statistically significant for all analyses. The calculations were performed with InStat 3.01 (GraphPad Software, San Diego, CA, USA) and SPSS 17.0 (IBM SPSS Software GmbH, Munich, Germany).

## RESULTS

A total of 275 patients with pT1 gastric adenocarcinoma were included in the study. LNM were present in 36 out of 275 (13.1%) patients. Gender distribution was 166 males to 109 females, with a median age of 64.0 years (range 32 to 85). Histologically, intestinal tumors according to the Lauren classification were predominant (53.6%) compared with diffuse (30.8%) and mixed type (15.6%). One hundred and sixty-two out of 275 tumors (59.0%) showed submucosal invasion (pT1b). Lymphovascular invasion (L1) was present in 23 out of 275 tumors (8.4%). Demographic, clinical and tumor characteristics of patients with and without LNM are listed in Table 1. In univariable analysis, patients with LNM were significantly younger, were more likely to be female and had significantly deeper tumor invasion. Tumors with LNM were significantly more likely to have a diffuse or mixed histology according to the Lauren classification and showed more often lymphovascular invasion. Nine out of 36 patients with LNM (25.0%) had lymphovascular invasion and all of them showed submucosal invasion.

**Table 1:** Univariable analysis of risk factors for lymph node metastasis in 275 patients undergoing surgical treatment for early gastric cancer

	LNM + (13%, *N*= 36)	LNM - (87%, *N* = 239)	ALL PATIENTS (*N* = 275)	ODDS Ratio, 95% CI	*p*-value
**Age (mean±SD)**	58.3 years (±13.4)	63.0 years (±11.1)	62.4 years (±11.5)		0.02^e^
**Gender**				**3.15 (1.52; 6.53)**	0.002^e^
Female (%)	23 (63.9%)	86 (36.0%)	109 (39.64%)		
Male (%)	13 (36.2%)	153 (64.0%)	166 (60.4%)		
**Depth of invasion^a^**				**5.34 (1.82; 15.67)**	0.001^e^
pT1a (%)	4 (11.1%)	94 (39.3%)	98 (35.6%)		
pT1b (%)	30 (83.3%)	132 (55.2%)	162 (58.9%)		
**Subgroups & tumor size^b^ (mean±SD)**					<0.001^f^
m1 (%), 0.85±0.21 cm	0 (0%)	2 (1.1%)	2 (1.0%)		
m2 (%), 2.66±2.53 cm	1 (3.7%)	46 (25.4%)	47 (22.6%)		
m3 (%), 2.69±1.77 cm	2 (7.4%)	25 (13.8%)	27 (13.0%)		
sm1 (%), 2.41±1.60 cm	7 (25.9%)	35 (19.3%)	42 (20.2%)		
sm2 (%), 2.72±1.78 cm	6 (22.2%)	37 (20.4%)	43 (20.7%)		
sm3 (%), 2.91±1.65 cm	11 (40.7%)	36 (19.9%)	47 (22.6%)		
**Tumor size^c^ (mean±SD)**	3.16 cm (±1.91)	2.66 cm (±1.87)	2.73 cm (±1.88)		0.170^e^
**Lauren subtype^d^**				**4.33 (1.87; 10.03)**	<0.001^g^
Diffuse (%)	10 (30.3%)	67 (30.9%)	77 (30.8%)		
Intestinal (%)	8 (24.2%)	126 (58.1%)	134 (53.6%)		
Mixed (%)	15 (45.5%)	24 (11.1%)	39 (15.6%)		
**Lymphovascular invasion**				**5.36 (2.12; 13.55)**	0.001^e^
Yes (%)	9 (25.0%)	14 (5.9%)	23 (8.4%)		
No (%)	27 (75.0%)	225 (94.1%)	252 (91.6%)		

a available in 260 patients

b available in 208 patients

c available in 223 patients

d available in 250 patients

e Fisher's exact test

f chi-square test for trend

g chi-square test

**Figure 1:**
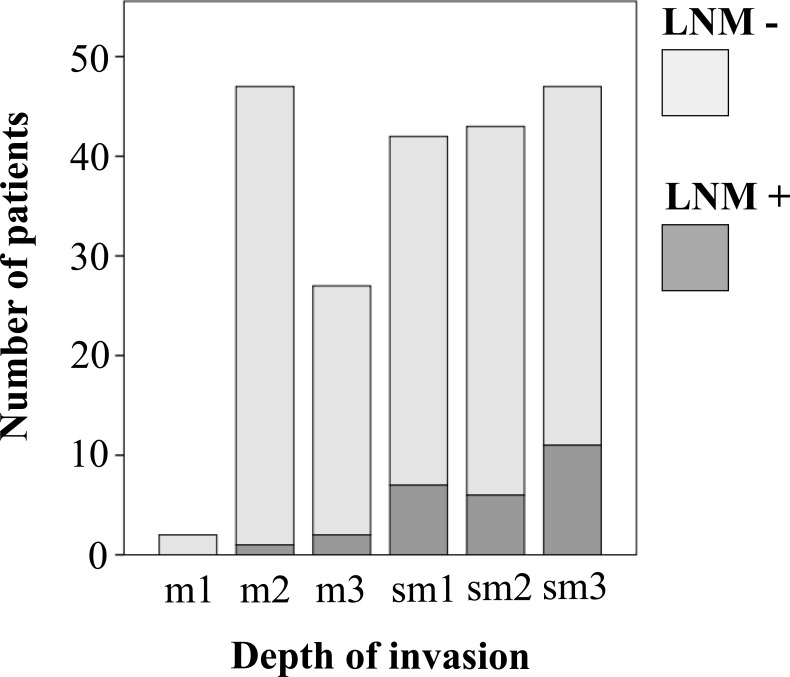
Distribution of lymph node metastases according to depth of invasion LN: lymph node; m1: invasion into upper third of mucosa (relation of LN-negative to LN-positive = 2:0; LN-positive: 0%); m2: invasion into middle third of mucosa (46:1; 2.1%); m3: invasion into lower third of mucosa (25:2; 7.4%); sm1: invasion into upper third of submucosa (35:7; 16.7%); sm2: invasion into middle third of submucosa (37:6; 14.0%); sm3: invasion into lower third of submucosa (36:11; 23.4%); chi-squared test for trend: *p* < 0.001.

Tumors with LNM were of larger diameter compared to those without LNM (3.16 cm (±1.91) *versus* 2.66 cm (±1.87)), but the difference failed to reach statistical significance (*p*-value: 0.170). There was a positive association of tumor diameter and invasion depth: whereas mean tumor diameter in m1 was only 0.85 cm, highest values were reached for sm3 with a mean diameter of 2.91 cm. LNM were significantly correlated with depth of invasion (*p*-value: 0.001). While no LNM were present in m1 tumors, 23.4% of sm3 EGC were nodal positive (Figure 1). With increasing invasion depth (from m1 to sm3) the proportion of patients with LNM increased (chi-squared test for trend, *p*-value < 0.001). When the submucosa was infiltrated (level sm1 or deeper), patients were almost five times more likely to have LNM than at mucosa levels (odds ratio: 4.9, 95% confidence interval: 1.19-19.97, *p*-value: 0.033). In the final logistic regression model, multivariable analysis revealed depth of invasion, lymphatic invasion, Lauren subtype and female gender as significant independent risk factors for LNM (Table 2).

**Table 2:** Multivariable analysis of risk factors for lymph node metastasis in 275 patients undergoing surgical treatment for early gastric cancer

	Odds ratio (95% confidence interval)
Depth of invasion (submucosa *versus* mucosa)	4.25 (1.35; 13.33)
Lymphovascular invasion (yes *versus* no)	4.01 (1.28; 12.58)
Lauren subtype (diffuse or mixed-type *versus* intestinal type)	3.14 (1.12; 8.83)
Gender (female *versus* male)	2.68 (1.14; 6.28)
Age (one-year difference)	0.97 (0.94; 1.01)

### Risk profile analysis

It is of particular interest to re-evaluate cases with LNM in the context of the current guidelines for endoscopic resection of EGC. In our population, the earliest stage of EGC presenting with LNM was a pT1a (m2) carcinoma of small size (1.2 cm) and intestinal type according to Lauren. In the next subgroup of EGC, namely the pT1a (m3) cases, two out 25 cases showed LNM. One carcinoma had intestinal histology and no additional histological risk factor. The second LNM positive carcinoma in the pT1a (m3) group presented with a size of 4.0 cm and mixed type histology according to Lauren. While lymphadenectomy would be recommended at least by some treatment guidelines for the latter case (due to the partly undifferentiated histology), the remaining two cases would not be considered aggressive since none of the risk parameters indicate a high risk for LNM.

Evaluating the group of pT1b (sm1) EGC, one can observe an obvious increase in LNM incidence from 3.9 % (m3) to 18.2 % (sm1), thereby generally questioning safe endoscopic treatment in this group. Multivariable logistic regression analyses also showed the significance of submucosa infiltration for predicting LNM. However, by taking into account additional risk factors (patients with diffuse or mixed histology and lymphovascular invasion) new aspects arise. In cases with intestinal type histology and without lymphovascular invasion (“low risk profile”), the risk of LNM is low (1/43 cases; 2.3%) when invasion depth is not beyond the sm2 level (Table 3).

**Table 3:** Lymph node metastases in patients with gastric cancer of intestinal type and lack of lymphovascular invasion (“low risk profile”) according to depth of invasion into the mucosa/submucosa

	No LNM	LNM
m1	1	0
m2	25	1
m3	12	1
sm1	22	1
sm2	21	0
sm3	15	2

## DISCUSSION

The aim of this study was to re-evaluate and verify the current guidelines for endoscopic treatment of EGC in a Western population by analysing histopathological predictors for LNM. To our knowledge, this study is the largest to date conducted in a Western population. After multivariable analysis three histopathological parameters, namely subtype according to the Lauren classification, lymphovascular invasion and depth of invasion, could be identified as independent predictors for LNM. Additional non-tissue-based factors like age and gender could also be identified as risk factors for LNM. Increasing tumor size was associated with LNM but failed to reach statistical significance.

In our multivariable regression analyses, Lauren classification was identified as an independent predictor for LNM. Diffuse-type and mixed-type histology showed an increased risk for the presence of LNM. This is in line with the findings of several studies demonstrating that diffuse type EGC had the highest rate of LNM [12, 20-23]. Conversely, Bamboat and colleagues found that diffuse type in early stages did not show an inferior prognosis compared with intestinal types of gastric cancer [25]. It should be noted that mixed-type histology in our study presented a particularly strong association with LNM, which confirms earlier findings that mixed type affects outcomes by a higher risk of LNM [26, 27]. The second histological parameter which was significant for predicting LNM in our study was lymphovascular invasion (L1). While this is in line with findings by Gotoda and colleagues [11], most Western studies had not assessed this histopathologic feature [12, 20-23]. Just recently our findings were confirmed by Ahmad et al. showing the importance of L1 in a small Western collective of EGC by multivariate analyses (*p* < 0.001) [19].

Despite high interobserver variability in diagnosis of lymphovascular invasion [28] many guidelines recognize the predictive importance of L1 and rule out endoscopic treatment for EGC with lymphatic invasion [6, 14, 16].

The strongest predictor of LNM in our analyses was depth of invasion. The current TNM classification separates pT1 gastric cancers into tumors invading the lamina propria or muscularis mucosae (pT1a) and those invading the submucosa (pT1b) [24]. This separation seems justified since we found a significant increase of LNM risk from pT1a to pT1b (3.9% in m3 *versus* 18.2% in sm1 EGC). Independently of this separation it is widely debated whether a certain depth of mucosal or submucosal invasion can serve as a cut-off criterion for the risk of LNM. Although some studies could not demonstrate a significant association between depth of invasion and LNM, the majority found a positive correlation [20-23, 29-31]. One of the few Western series available stated that endoscopic procedures should be limited to m1 and m2 carcinomas on the basis of an observed rate of 13% LNM in m3 carcinomas [12]. However, this is in contrast to the findings by Ahmad and colleagues also examining Western EGC patients and detecting only one LNM positive patient out of 23 pT1a tumours [19]. The finding is in line with many other publications finding a relevant incidence of LNM only in tumors with submucosal invasion (pT1b), advocating that all pT1a carcinomas are suitable for endoscopic treatment [7, 17, 18, 24].

In our study, LNM were present in only two pT1a (m3) cases, but this still accounted for a LNM risk of 7.4% because of the small total number of patients in this group. This makes conclusions for this subgroup difficult and draws attention to the pT1b (sm1) group (42 cases in total, 7 LNM positive). In this group, there is a substantial increase in the rate of LNM (16.7% *versus* 7.4% in m3) which is mirrored by significance of the comparison of submucosal with mucosal invasion in the multivariable logistic regression analysis. However, when considering additional histological risk factors (excluding patients with diffuse or mixed histology or lymphovascular invasion; “low risk profile”) only one case with LNM remained in the pT1b (sm1) group. This finding was even more pronounced in the pT1b (sm2) group: after exclusion of cases with diffuse or mixed histology and lymphovascular invasion no case with LNM remained. In the pT1b (sm3) group, in turn, the LNM risk was higher even when the other histopathological factors matched a “low risk profile”. While this “case by case” discussion by considering additional histological risk factors can be helpful for decision making in selected patients it does not give a clear rationale for endoscopic treatment in pT1b EGCs. Since the majority of studies, including ours, indicate LNM in up to 20% of patients with pT1b tumors, surgical resection with lymphadenectomy remains the gold standard. The additional histological factors cannot be disregarded, but since these are based on low absolute patient numbers, “extended criteria” for endoscopic resection should only be defined in the context of prospective large multicenter studies.

Another non-tissue-based factor statistically associated with increased risk of LNM was female gender. Albeit no convincing biological rationale can be given for this observation, a previous study did report the same phenomenon in a Japanese population [32]. In clinical routine and the current guidelines, gender does not play a relevant role for quantifying the risk of LNM and deciding on this basis about endoscopic therapy in patients with EGC, and further research is needed to corroborate this finding.

Our study has a number of methodological limitations. First, it is a retrospective series from a single centre covering a large time period and the findings need external validation in prospective studies. Second, surgical and histopathological techniques have not been standardized and are prone to variation over time. Although for most of the specimens histopathological reassessment by experienced gastrointestinal pathologists was possible, a number of cases had to be included based on the original pathological reports, where some data were incomplete. In particular, we lacked information on ulceration and could not include this aspect in our analyses although it has been shown to be a risk factor for LNM in other series. Third, although systematic lymphadenectomy was the institutional standard for the entire study period, its true extent remains unclear as for many patients information on the exact number of harvested lymph nodes was not available. This is important, as the risk of LNM might be underestimated in cases with incomplete lymphadenectomy, because metastatic lymph nodes could have been missed during surgery. Fourth, although our cohort represents one of the largest non-Asian series published, the absolute number of patients with LNM is still relatively low. This limits the statistical power of our analyses and thus some true predictors of LNM might not have been identified. Additionally, the study simplifies the complex problem of LNM. Unfortunately, not all patients are cured by removal of LNM, but rather develop distant metastases subsequently, which ultimately leads to a fatal outcome. The study does not take into account that these patients do not benefit from lymphadenectomy. For reasons of clarity and rationalization, it was assumed that in the case of LNM patients will benefit from lymphadenectomy.

In conclusion, we were able to identify significant independent risk factors for LNM in EGC in a large Western cohort. Our model identified known parameters like lymphovascular invasion and depth of invasion. We were also able to demonstrate that the Lauren classification, which has existed for a long time, is still useful in predicting LNM in EGC by recognizing diffuse and mixed histology as a risk factor. We showed that submucosal invasion is a major risk factor for LNM in EGC and should lead to the recommendation of gastrectomy with lymphadenectomy. The possibility of performing endoscopic treatment in pT1b EGC in the absence of histological factors associated with high LNM risk should be evaluated in a clearly defined prospective clinical trial.

## References

[1] Ferlay J, Steliarova-Foucher E, Lortet-Tieulent J, Rosso S, Coebergh JW, Comber H, Forman D, Bray F (2013). Cancer incidence and mortality patterns in Europe: estimates for 40 countries in 2012. Eur J Cancer.

[2] Aaltonen L, Hamilton S (2000). Pathology and Genetics of Tumours of the Digestive System.

[3] Green PH, O'Toole KM, Weinberg LM, Goldfarb JP (1981). Early gastric cancer. Gastroenterology.

[4] Quiros RM, Bui CL (2009). Multidisciplinary approach to esophageal and gastric cancer. Surg Clin North Am.

[5] Takeshita K, Tani M, Inoue H, Saeki I, Hayashi S, Honda T, Kando F, Saito N, Endo M (1997). Endoscopic treatment of early oesophageal or gastric cancer. Gut.

[6] Moehler M, Al-Batran SE, Andus T, Anthuber M, Arends J, Arnold D, Aust D, Baier P, Baretton G, Bernhardt J, Boeing H, Bohle E, Bokemeyer C (2011). [German S3-guideline “Diagnosis and treatment of esophagogastric cancer”]. Z Gastroenterol.

[7] Alvarez HL, Pouw RE, van Vilsteren FG, Ten Kate FJ, Visser M, van Berge Henegouwen MI, Weusten BL, Bergman JJ (2010). Risk of lymph node metastasis associated with deeper invasion by early adenocarcinoma of the esophagus and cardia: study based on endoscopic resection specimens. Endoscopy.

[8] Kienle P, Buhl K, Kuntz C, Dux M, Hartmann C, Axel B, Herfarth C, Lehnert T (2002). Prospective comparison of endoscopy, endosonography and computed tomography for staging of tumours of the oesophagus and gastric cardia. Digestion.

[9] Kim SK, Kang KW, Lee JS, Kim HK, Chang HJ, Choi JY, Lee JH, Ryu KW, Kim YW, Bae JM (2006). Assessment of lymph node metastases using 18F-FDG PET in patients with advanced gastric cancer. Eur J Nucl Med Mol Imaging.

[10] Willis S, Truong S, Gribnitz S, Fass J, Schumpelick V (2000). Endoscopic ultrasonography in the preoperative staging of gastric cancer: accuracy and impact on surgical therapy. Surg Endosc.

[11] Gotoda T, Yanagisawa A, Sasako M, Ono H, Nakanishi Y, Shimoda T, Kato Y (2000). Incidence of lymph node metastasis from early gastric cancer: estimation with a large number of cases at two large centers. Gastric Cancer.

[12] Holscher AH, Drebber U, Monig SP, Schulte C, Vallbohmer D, Bollschweiler E (2009). Early gastric cancer: lymph node metastasis starts with deep mucosal infiltration. Ann Surg.

[13] Shen L, Huang Y, Sun M, Xu H, Wei W, Wu W (2009). Clinicopathological features associated with lymph node metastasis in early gastric cancer: analysis of a single-institution experience in China. Can J Gastroenterol.

[14] (2015). NCCN Clinical Practice Guidelines in Oncology - Gastric Cancer.

[15] Waddell T, Verheij M, Allum W, Cunningham D, Cervantes A, Arnold D (2014). Gastric cancer: ESMO-ESSO-ESTRO clinical practice guidelines for diagnosis, treatment and follow-up. Eur J Surg Oncol.

[16] (2011). Japanese gastric cancer treatment guidelines 2010 (ver. 3). Gastric Cancer.

[17] Soetikno R, Kaltenbach T, Yeh R, Gotoda T (2005). Endoscopic mucosal resection for early cancers of the upper gastrointestinal tract. J Clin Oncol.

[18] Tada M, Tanaka Y, Matsuo N, Shimamura T, Yamaguchi K (2000). Mucosectomy for gastric cancer: current status in Japan. J Gastroenterol Hepatol.

[19] Ahmad R, Setia N, Schmidt BH, Hong TS, Wo JY, Kwak EL, Rattner DW, Lauwers GY, Mullen JT (2015). Predictors of Lymph Node Metastasis in Western Early Gastric Cancer. J Gastrointest Surg.

[20] Folli S, Dente M, Dell'Amore D, Gaudio M, Nanni O, Saragoni L, Vio A (1995). Early gastric cancer: prognostic factors in 223 patients. Br J Surg.

[21] Saragoni L, Morgagni P, Gardini A, Marfisi C, Vittimberga G, Garcea D, Scarpi E (2013). Early gastric cancer: diagnosis, staging, and clinical impact. Evaluation of 530 patients. New elements for an updated definition and classification. Gastric Cancer.

[22] Popiela T, Kulig J, Kolodziejczyk P, Sierzega M (2002). Long-term results of surgery for early gastric cancer. Br J Surg.

[23] Roviello F, Rossi S, Marrelli D, Pedrazzani C, Corso G, Vindigni C, Morgagni P, Saragoni L, De MG, Tomezzoli A (2006). Number of lymph node metastases and its prognostic significance in early gastric cancer: a multicenter Italian study. J Surg Oncol.

[24] Sobin LH, Gospodarowicz MK, Wittekind C (2009). UICC: TNM classification of malignant tumors.

[25] Bamboat ZM, Tang LH, Vinuela E, Kuk D, Gonen M, Shah MA, Brennan MF, Coit DG, Strong VE (2014). Stage-stratified prognosis of signet ring cell histology in patients undergoing curative resection for gastric adenocarcinoma. Ann Surg Oncol.

[26] Hanaoka N, Tanabe S, Mikami T, Okayasu I, Saigenji K (2009). Mixed-histologic-type submucosal invasive gastric cancer as a risk factor for lymph node metastasis: feasibility of endoscopic submucosal dissection. Endoscopy.

[27] Nakata K, Nagai E, Miyasaka Y, Ohuchida K, Ohtsuka T, Toma H, Hirahashi M, Aishima S, Oda Y, Tanaka M (2012). The risk of lymph node metastasis in mucosal gastric carcinoma: especially for a mixture of differentiated and undifferentiated adenocarcinoma. Hepatogastroenterology.

[28] Harris EI, Lewin DN, Wang HL, Lauwers GY, Srivastava A, Shyr Y, Shakhtour B, Revetta F, Washington MK (2008). Lymphovascular invasion in colorectal cancer: an interobserver variability study. Am J Surg Pathol.

[29] Park DJ, Lee HK, Lee HJ, Lee HS, Kim WH, Yang HK, Lee KU, Choe KJ (2004). Lymph node metastasis in early gastric cancer with submucosal invasion: feasibility of minimally invasive surgery. World J Gastroenterol.

[30] Ishigami S, Hokita S, Natsugoe S, Tokushige M, Saihara T, Iwashige H, Aridome K, Aikou T (1998). Carcinomatous infiltration into the submucosa as a predictor of lymph node involvement in early gastric cancer. World J Surg.

[31] Kurihara N, Kubota T, Otani Y, Ohgami M, Kumai K, Sugiura H, Kitajima M (1998). Lymph node metastasis of early gastric cancer with submucosal invasion. Br J Surg.

[32] Kwee RM, Kwee TC (2008). Predicting lymph node status in early gastric cancer. Gastric Cancer.

